# *In vitro* in-depth characterization of emodepside in combination with key anthelmintic drugs on soil-transmitted helminths

**DOI:** 10.1128/aac.00825-26

**Published:** 2026-10-08

**Authors:** Anastasia Schärer, Cécile Häberli, Dominique Schlienger, Jennifer Keiser

**Affiliations:** 1 Department of Research, Swiss Tropical and Public Health Institute 30247, Allschwil, Switzerland; 2 University of Basel 27209, Basel, Switzerland; Houston Methodist Hospital and Weill Cornell Medical College, Houston, Texas, USA

**Keywords:** soil-transmitted helminths, drug combinations, emodepside

## Abstract

Despite the enormous disease burden, treatments are limited for soil-transmitted helminthiasis. The handful of anthelmintic drugs suffer from variable and often insufficient efficacy against the pathogens. Resistance development, caused by high drug pressure, is an ongoing concern. New treatments are needed, and combination therapies may represent a promising therapeutic strategy. We explored emodepside as a partner in combination therapy and characterized drug-interaction effects with key anthelmintics *in vitro* against different soil-transmitted helminths (STHs). Drug-interaction studies were carried out in human recombinant cytochromes P450 assays. Combination studies revealed species-, stage-, and drug-dependent interaction profiles. For benzimidazole-emodepside combinations, potentiation was only observed against *Ancylostoma ceylanicum* larvae, while against adult stages, up to threefold potentiation or synergy (ΣFIC < 0.9) was observed for *Heligmosomoides polygyrus* and *Trichuris muris*, especially for albendazole and albendazole sulfoxide. Combinations of emodepside with macrocyclic lactones showed mostly strong synergy against *H. polygyrus* larvae (ΣFIC 0.06–2.52), while other species trended toward antagonism (ΣFIC >1.5). Against adult stages, potentiation or synergism was observed in *H. polygyrus* and *T. muris*, while against *Strongyloides ratti*, only the moxidectin-emodepside combination was synergistic (ΣFIC 0.69–0.99). For nAChR agonists, levamisole showed the strongest effect against *Necator americanus* larvae, while oxantel pamoate exhibited potentiation or synergistic effects across multiple species and life stages. CYP450 inhibition assays reveal no relevant drug-drug interactions. This study provides a thorough evaluation of emodepside as a combination partner against soil-transmitted helminths *in vitro*, paving the way for *in vivo* studies and ultimately a novel combination therapy with emodepside against STH infections.

## INTRODUCTION

Soil-transmitted helminthiasis is a widespread neglected tropical disease (NTD) that affects around one-quarter of the world’s population, mainly in subtropical and tropical areas (1–3). Soil-transmitted helminth (STH) infections are associated with insufficient resources, more precisely clean drinking water, sanitation, and hygiene (4, 5). The disease-causing agents are the gut-dwelling STHs, *Ascaris lumbricoides* (roundworm), *Trichuris trichiura* (whipworm), and *Necator americanus* or *Ancylostoma duodenale* (hookworms) (1).

At present, the control of these infections is achieved through regular and broad distributions of one of the two benzimidazoles, albendazole or mebendazole, to at-risk populations in endemic areas (2, 6). However, both drugs exhibit distinct and insufficient treatment efficacies, especially against hookworm and *T. trichiura* infections (7). In more detail, mebendazole shows low cure rates (CR, based on 95% confidence interval [CI]) for hookworm (20.8%–46.9%) and *T. trichiura* (25.9%–60.2%), while albendazole shows better efficacy against hookworm (71.5%–85.6%) but remains poor for *T. trichiura* (21.0%–42.5%) (7).

*Strongyloides stercoralis* (threadworm) is also considered an STH, although it does not share the same characteristics as the other species, particularly with regard to its clinical identification and treatment requirements (1, 4, 8). While being neglected for many years, in 2024, the WHO developed guidelines on a public health strategy for strongyloidiasis with the goal of establishing an effective control program in endemic settings by 2030 (1, 9–11).

As patients with *S. stercoralis* infection are less responsive to albendazole (12), the standard of care against strongyloidiasis is the macrocyclic lactone, ivermectin (1, 13). Despite the high efficacy against *S. stercoralis* (CR 88%) (14), ivermectin is contraindicated in *Loa loa*-endemic areas, as it can cause severe neurological adverse events and must be dosed according to body weight, posing logistical challenges for large-scale administration (15–17). Moreover, unlike albendazole and mebendazole, which are donated for the treatment of STH infections, ivermectin is only available through drug donation programs targeting large-scale control of lymphatic filariasis and onchocerciasis (1, 18). Furthermore, the risk of developing drug resistance, driven by high drug pressure associated with large-scale distribution of these drugs, is an ongoing concern, as it has already been widely documented in veterinary medicine (19, 20).

Drug repurposing is a common approach in the field of NTDs. Many of the currently approved drugs against NTDs were once developed for another disease, application, or field of interest, like agriculture or veterinary medicine (21). Emodepside was repurposed from the veterinary field and explored (pre-clinically and clinically) also as a candidate to treat human hookworm and *T. trichiura* infections. Clinical trials reported observed CRs of 81% (95% CI: 58.0%–95.0%) against hookworm, 100% (95% CI: 69.2%–100%) against *A. lumbricoides*, 92% (95% CI: 74.0%–99.0%) against *S. stercoralis*, and 100% (95% CI: 88.0%–100%) against *T. trichiura* infections, at a 15 mg dose (22–25). Despite the remarkable activity of emodepside as monotherapy, combining emodepside with another anthelmintic drug could increase the drug’s efficacy and delay possible resistance evolution (26–28). A prominent example of combination chemotherapy for STH infections is albendazole-ivermectin, which has shown superior efficacy against *T. trichiura* infections compared to albendazole alone (29).

The aim of this study was to conduct an in-depth characterization of combinations with emodepside and selected key anthelmintics (benzimidazoles and macrocyclic lactones, as well as other commercially available anthelmintics [30]) against different species and developmental stages of STHs *in vitro*.

As co-administration of two or more drugs can influence the activity of drug-metabolizing enzymes, we additionally assessed drug-drug interaction (DDI) of the same combinations against CYP450 enzymes using validated fluorescence assays.

## MATERIALS AND METHODS

### Ethics and animals

Female mice (C57BL/6NRj strain, 3 weeks) and male golden Syrian hamsters (RjHan:AURA strain, 3 weeks) were purchased from Janvier Laboratories (Le Genest-Saint-Isle, France). Female mice (NMRI strain, 3 weeks) were purchased from Charles River (Sulzfeld, Germany). All rodents were housed in IVC cages supplied with soft wood chip bedding, chewing wood, and free access to water and food. The cages were kept in a room with controlled conditions (temperature 22°C–24°C, relative humidity 45%–65%, artificial lighting with a circadian cycle of 12 h of light [6 a.m.–6 p.m.]). Rodents were acclimatized for 1 week before infection.

### Compounds and culture media

Albendazole, ivermectin, levamisole hydrochloride, oxantel pamoate, pyrantel pamoate, and albendazole sulfoxide were the products of Sigma-Aldrich (Buchs, Switzerland). Mebendazole was acquired from Laboratoria Wolfs (Zwijndrecht, Belgium). Moxidectin was purchased from Hangzhou Dingyan Chem. Co., Ltd. (Hangzhou, China). Tribendimidine was obtained from Shandong Xinhua Pharmaceutical Company Limited (Zibo, China). The compounds were dissolved in pure dimethyl sulfoxide (DMSO 100%, Sigma-Aldrich, Switzerland) at a concentration of 10 mM, aliquoted, and stored at −20°C until further use. Ketoconazole, omeprazole, α-naphthoflavone hydrochloride, and quinidine, which were used as positive controls for CYP450 enzymes in fluorescence-based assays, were also obtained from Sigma-Aldrich (Buchs, Switzerland).

*Trichuris muris* larvae (L1) and larvae (L3) and adult worms of *Heligmosomoides polygyrus* were cultured in RPMI 1640 medium (Gibco, Waltham, MA, USA) supplemented with 1% (vol/vol) penicillin/streptomycin mix solution (10,000 U/mL/10 mg/mL, Sigma-Aldrich, Buchs, Switzerland), and 5% (vol/vol) amphotericin B (250 μg/mL, Sigma-Aldrich, Buchs, Switzerland). For adult *T. muris*, the same culture medium was supplemented with 1% (vol/vol) penicillin/streptomycin solution and 5% (vol/vol) fetal calf serum (FCS, Bioconcept, Allschwil, Switzerland). Larvae (L3) of *Necator americanus* and *Ancylostoma ceylanicum* were cultured in Hanks’ Balanced Salt Solution (Gibco, Waltham, MA, USA) supplemented with 1% (vol/vol) penicillin/streptomycin and 5% (vol/vol) amphotericin B. For *Strongyloides ratti* larvae (L3), phosphate-buffered saline (PBS 1×; Sigma-Aldrich, Buchs, Switzerland) was used as the culture medium and supplemented with 1% (vol/vol) penicillin/streptomycin solution. For adult *S. ratti,* RPMI was supplemented with 1% (vol/vol) penicillin/streptomycin solution and 5% (vol/vol) horse serum (Thermo Fisher Scientific, Allschwil, Switzerland).

To hatch *T. muris* eggs and harvest the L1, the RPMI 1640 culture medium was supplemented with 1% (vol/vol) penicillin/streptomycin solution, 5% (vol/vol) amphotericin B, and 20% (vol/vol) of FCS. For *Escherichia coli* (DSM 30083), which was utilized to initiate the hatching process, Luria broth medium (LB) was prepared by dissolving 1 g /50 mL dehydrated medium concentrate (Thermo Fisher Scientific, Allschwil, Switzerland) in purified water (Milli-Q).

All media were sterilized by filtration using a 0.22 μm filter bottle (Vitaris AG, Baar, Switzerland) under laminar flow.

### Parasites and bacteria

The life cycles of *T. muris, H. polygyrus*, *N. americanus*, *A. ceylanicum,* and *S. ratti* are established and maintained at the Swiss TPH (30).

#### Larvae harvesting

To obtain *T. muris* eggs, female C57BL/6NRj mice were infected per os with 200–250 eggs showing around 90%–95% embryonation (confirmed microscopically). After 41 days post-infection (p.i.), unembryonated eggs were collected by filtering and centrifuging the feces of infected mice. After isolation, eggs were stored in purified water (Milli-Q) at room temperature (RT) in the dark for at least 3 months to allow the complete embryonation process. For the drug sensitivity *in vitro* assays, *T. muris* L1 were hatched by preparing a 2 mL egg suspension containing 2,000 eggs (1 egg/µL) and freshly prepared culturing media. The eggs were then washed in hatching medium by centrifuging the suspension at RT at 805 rcf for 10 min, after which 500 µL of the medium was replaced with 500 µL (~6.3 × 10^8^ cells) of an *E. coli* suspension. The eggs and bacterial suspension were subsequently incubated for at least 4 h at 37°C and 5% CO_2_, and hatching rates of 50%–60% were considered acceptable.

To obtain *H. polygyrus* eggs, female NMRI mice were infected per os with ~150 L3 showing a survival rate of 80%–90%. After 16 days p.i., unembryonated eggs were isolated by filtering and centrifuging the feces of the infected mice. The isolated eggs were transferred onto prepared agar plates and incubated for 7 days at RT in a dark and humid environment to allow embryonation, hatching, and development to the L3 stage.

To obtain *N. americanus* or *A. ceylanicum* eggs, male RjHan:AURA hamsters were infected by subcutaneous injection of 150 L3 or 140 L3, respectively. At 35 days p.i., unembryonated eggs were isolated by filtering and centrifuging the feces from infected hamsters, then transferred on prepared agar plates. The plates were incubated under the same conditions as *H. polygyrus*. The isolated larvae were transferred after agar plate washing into 20 mL plastic tubes and stored in the dark at RT.

For *S. ratti,* 3-week-old male Wistar rats (Janvier Laboratories, Le Genest-Saint-Isle, France) were infected by subcutaneous injection of 1,300 *S. ratti* L3. Two weeks p.i., feces were collected, and L3 were isolated using the Baermann technique, as described elsewhere (31). Briefly, collected feces were placed on a mesh within a funnel with activated charcoal. Warm water was added to stimulate larvae to migrate downward, and the apparatus was incubated for 24–48 h. The isolated larvae were transferred into culture medium within 30 min of harvesting and stored at 4°C–8°C.

#### Adult worm harvesting

Adult *T. muris* and *H. polygyrus* worms were collected by dissecting and manually removing the adult worms from the intestinal wall of sacrificed mice 41 and 14 days p.i., respectively. Worms were transferred to petri dishes containing adult worm culture media. Adult *S. ratti* worms were collected 21 days p.i. by dissecting, removing, and immersing the sacrificed rat’s intestine in the culture medium. The intestines were incubated for 4 h at 37°C and 5% CO_2_ to allow the adult worms to detach from the intestinal wall. Due to ethical considerations, as hamsters harbor only a low number of worms (<10) per animal, *in vitro* drug sensitivity assays were not performed on adult *N. americanus* and *A. ceylanicum* .

#### Bacteria

*Escherichia coli* (DSM 30083) was obtained as freeze-dried pellets from the German Collection of Microorganisms and Cell Cultures (Leibniz Institute DSMZ, Braunschweig, Germany). The bacterial culture was prepared under aseptic conditions as described previously (32). The overnight culture was initiated by transferring 50 µL of the glycerol stock into 5 mL of freshly prepared LB medium, vortexing briefly, and incubating overnight at 37°C and 5% CO_2_.

### *In vitro* phenotypic drug sensitivity assays

For the *T. muris* L1 assay, 50 µL containing 20–30 freshly hatched larvae were suspended in wells containing culture media and either a single drug, a drug combination, or a negative control (1% DMSO) to reach a final volume of 100 µL in sterile 96-well flat-bottom plates (Ref. Nr.: 353072, Corning, USA). To ensure uniform distribution and quantity, the larvae were counted under an inverted transmitted-light microscope at 10× magnification (Axiovert 40 CFL, Carl Zeiss, Germany). The plates were incubated at 37°C and 5% CO_2_ for 24 h.

For *H. polygyrus* L3, *N. americanus* L3, *A. ceylanicum* L3, and *S. ratti* L3 drug sensitivity assays, 50 µL containing 30–40 larvae were transferred into wells containing culture media and either a single drug, a drug combination, or a negative control (1% DMSO) to reach the final volume of 250 µL in sterile 96-well flat-bottom plates. The larval count was verified as above. The plates were sealed with Parafilm and incubated at RT in the dark for 72 h.

To assess drug effects, the number of motile L1/L3 was quantified under an inverted transmitted-light microscope at 2.5–4× magnification after activation with 80 µL of ~80°C hot water.

For the adult drug sensitivity assays, three to four adult worms of *T. muris* or *H. polygyrus* were transferred into wells containing culture media and either a single drug, a drug combination, or a negative control (1% DMSO) in a final volume of 2 mL. The adult worms were incubated at 37°C and 5% CO_2_ and examined after 24 h, 48 h, and 72 h of drug exposure. Scoring was performed on a scale of 0–3 with quarter intervals, based on appearance, egg production, and motility, under an inverted transmitted-light microscope at 10–20× magnification.

#### Half maximal effective concentration (EC_50_) determination

For larval assays, EC_50_ values were determined by exposing the larvae to a total of eight drug concentrations, starting at 50 and 10 µM, prepared from two 10-fold serial dilutions. Each concentration was tested in triplicates, and all assays were performed in at least two independent experiments. Due to the limited availability of *H. polygyrus*, *T. muris,* and *S. ratti* adult worms, EC_50_ determination was not performed for the adult stage. Instead, previously determined EC₅₀ values from adult-stage assays were used as a starting point for the combination assays (22, 30).

#### Drug combination assays

**Fig 1:**
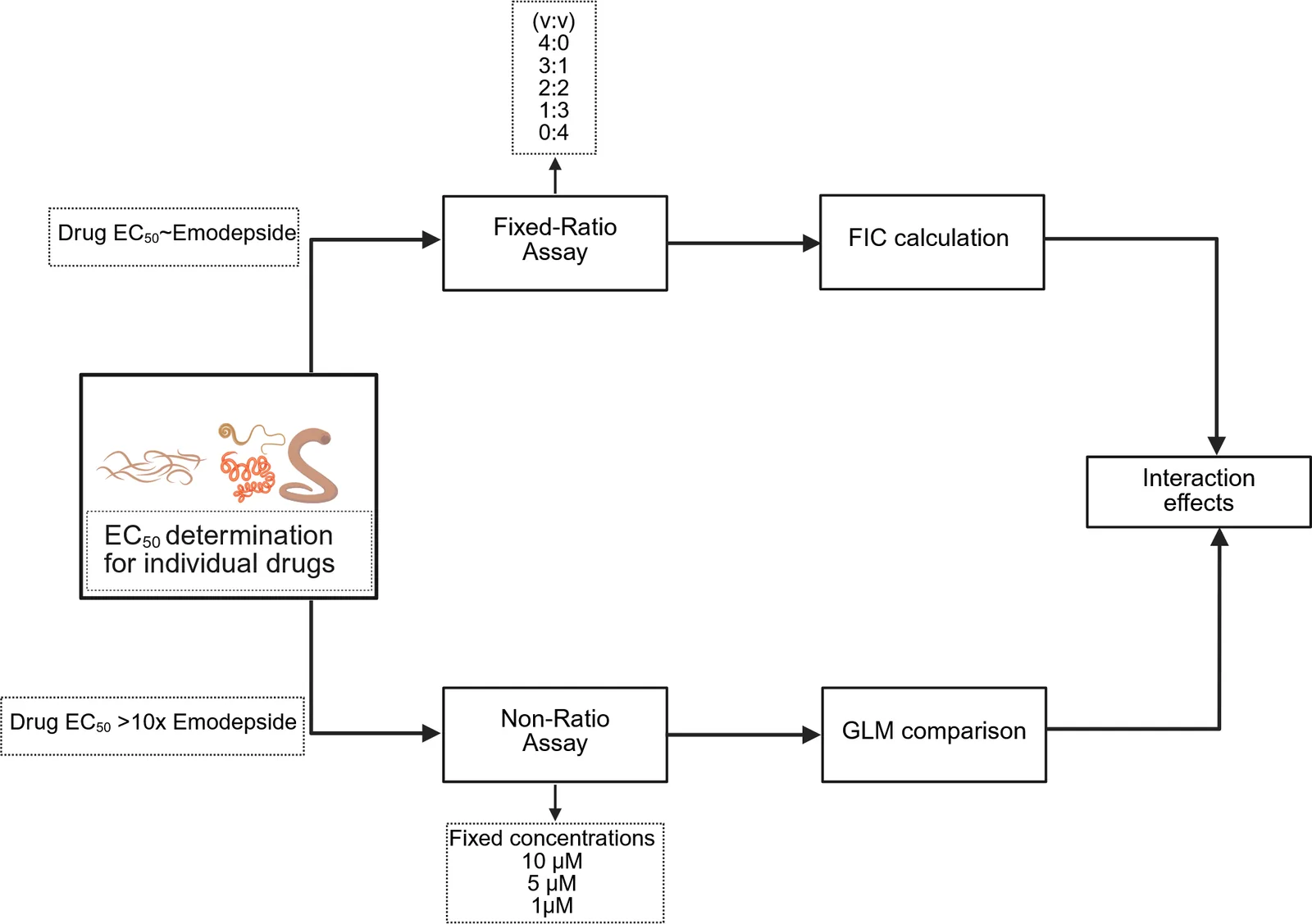
Drug combination evaluation study design.

To evaluate potential synergistic, additive, or antagonistic effects, emodepside was tested in combination with selected commercially available anthelmintics (Fig. 1).

For compounds that were inactive (EC_50_ >100 µM) or exhibited an EC_50_ >10-fold (except for *T. muris*) higher than that of emodepside, culture medium was prepared containing the compound at 1, 5, or 10 µM, to which the emodepside dilution series, as described above, was added.

For compounds with EC_50_ values similar to that of emodepside, drug combinations were prepared by first generating stock solutions in culture media of each compound at 10× its predetermined EC_50_ concentration. The two drugs were then combined at the following (vol/vol) ratios: 4:0, 3:1, 2:2, 1:3, and 0:4. For larval assays, eight concentrations were tested per ratio, while for adult worm assays, five concentrations were tested per ratio using a twofold serial dilution.

### *In vitro* CYP450 DDI assay

Potential DDI between emodepside and the other test compounds was assessed on CYPs commonly involved in drug metabolism, namely CYP1A2, CYP2C9, CYP2C19, CYP2D6, and CYP3A4. The corresponding Vivid CYP450 Screening Kits (Thermo Fisher Scientific, Allschwil, Switzerland) were used following the manufacturer’s instructions (33).

For both single compounds and drug combinations, 3.16-fold serial dilutions were prepared to achieve final concentrations of 100, 31.6, 10, 3.16, 1, 0.316, and 0.10 µM. Positive controls consisted of CYP-specific inhibitors, α-naphthoflavone (CYP1A2), omeprazole (CYP2C19), diclofenac (CYP2C9), ketoconazole (CYP3A4), and quinidine (CYP2D6). The concentrations for propranolol, omeprazole, and diclofenac were the same as for the single and combined drugs. For quinidine and ketoconazole, the concentrations were adjusted to the initial assay concentrations of 10, 3.16, 1, 0.316, 0.1, 0.0316, and 0.01 μM. The negative control consisted of CYPs exposed to 2% DMSO.

Working dilutions of single drugs, drug combinations, or positive controls and negative controls were transferred into black 96-well plates (Ref. Nr.: 237108, Thermo Fisher Scientific, Allschwil, Switzerland). Baculosomes containing the human recombinant CYPs were mixed with the Vivid regeneration system and reaction buffer and were added to the wells. The plates were incubated at RT for 10 min, during which the background fluorescence was recorded using a SpectraMax M2 plate reader (Molecular Devices, Softmax, version 5.4.6).

Subsequently, the fluorogenic CYP substrate and nicotinamide adenine dinucleotide phosphate (NADP+) were added to the wells to initiate the reaction. After 2 min, fluorescence was measured at 1 min intervals for 60 min. The specific reaction time for each CYP was selected based on the linear phase of metabolite formation, as described previously (34). The assays were performed in duplicate and repeated once.

### Data analysis

*In vitro* drug activity was determined by normalizing motile larval counts in treated wells compared to the counts of motile larvae of the controls and averaging across replicates. For adult worms, viability was scored on a 0–3 scale and normalized to controls. EC_50_ values were estimated through application of the non-linear least-squares regression using a four-parameter (sigmoid) function. All curve fitting and EC_50_ value determinations were performed with R (version 4.5.1) (35) as previously described (36).

For each drug ratio, fractional inhibitory concentration (FIC) values were calculated by dividing EC_50_ values of each drug in the combination by the EC_50_ of the drug alone. The sum of the two FIC values (ΣFIC) was then used to assign the interaction type. Antagonism was defined as ΣFIC >1.5, additivity was defined as ΣFIC = 0.9–1.5, and synergy was defined as ΣFIC <0.9 (37, 38). Drug interactions were assessed using isobologram analysis based on FIC values and the between-experiment standard error of the mean. All visualizations were generated in R using the ggplot2 package. For combination studies in which emodepside was administered together with drugs that were inactive *in vitro* or had a 10-fold higher EC_50_, generalized linear models (GLMs) with a binomial distribution (larval survival) or linear mixed-effects regression models (LMER) with a Gaussian distribution (adult worm viability scores) were used to compare outcomes between emodepside alone and emodepside in combination at fixed drug concentrations (1, 5, or 10 µM). The proportion of surviving larvae or adult viability score was modeled using the logit link or identity function, with treatment group, emodepside concentration, their interaction, and experimental cycle included as model terms. Pairwise comparisons between emodepside alone and each combination at each emodepside concentration were performed using estimated marginal means. Analyses focused on emodepside concentrations at which sufficient variation in response permitted meaningful comparison (0.05 µM–1.0 µM in larvae, 0.08 µM–0.02 µM in adult *H. polygyrus*, and 0.5 µM–0.1 µM in adult *S. ratti*). Predicted survival probabilities (larvae) or viability scores (adult worms) with 95% confidence intervals were estimated from the model and plotted across emodepside concentrations to visualize combination effects. All analyses were performed in R using lme4, emmeans, and ggeffects packages.

For CYP450 inhibition assays, background fluorescence was subtracted from the assay fluorescence following reaction initiation with NADP+. Inhibition was quantified by comparing the fluorescence values of the test compound with those of the corresponding DMSO controls. Drug inhibitory (IC_50_) values for the single drugs and combinations were determined by non-linear least-squares regression using a four-parameter logistic model. Consistent with the analysis applied for EC_50_ estimation, curve fitting and IC₅₀ determinations were performed and visualized in R. Percent inhibition was derived from normalized fluorescence relative to the minimum- and maximum-signal controls. The data were analyzed by a linear model containing treatment, concentration (categorical), and their interaction; the experimental run was included as a blocking factor. Each combination was compared to each single compound (where applicable) at each tested concentration using Dunnett-adjusted contrasts (α = 0.05). All analyses were performed in R using emmeans, and ggeffects packages.

## RESULTS

### *In vitro* activity of single drugs

Prior to the drug combination screening, we determined the EC_50_ for all selected drugs against the larval stage. Due to the limited availability of adult worms, we only performed an EC_50_ determination for emodepside. For other anthelmintics, published EC_50_ values (determined at 72 h) from our group were used as reference points for drug combination studies. The individual values are summarized in Tables S1 and S2.

All tested benzimidazoles showed no activity (EC_50_ >100 µM) against all larval species. Macrocyclic lactones exhibited variable potency across the individual larval species. We observed activity (EC_50_ <0.5 µM) against both hookworms (*N. americanus* and *A. ceylanicum*) L3 and *S. ratti* L3, while no activity (EC_50_ >100 µM) was observed against *T. muris* L1. Against *H. polygyrus* L3, the murine hookworm model, only moxidectin exhibited high activity (EC_50_ <0.5 µM), whereas abamectin and ivermectin showed low activity (EC_50_ >10 µM). nAChR agonists, such as levamisole, oxantel pamoate, pyrantel pamoate, and tribendimidine, exhibited variable potencies, ranging from no to high activity across all larval species (Table S1). In contrast, emodepside was the only drug that showed broad anthelmintic activity across all tested larval species, ranging from moderate (EC_50_ = 2.0 ± 0.5 µM against *A. ceylanicum*) to high activity (EC_50_ = 0.3 ± 0.04 µM against *T. muris*).

We assessed emodepside’s activity over 72 h against adult stages of selected species (Table S2). No time-dependent changes were observed. Emodepside demonstrated high potency against *T. muris* adults (EC_50_ = 0.01 ± 0.006 µM) and *H. polygyrus* and *S. ratti* (EC_50_ = 0.1 ± 0.01 µM and EC_50_ = 0.3 ± 0.04 µM, respectively).

### Fixed-ratio drug combinations

Tables 1 and 2 summarize the ΣFIC of the individual drug combinations and ratios against larval and adult stages. For adults, ΣFIC values calculated at additional time points are summarized in Table S6. The individual drug interactions for each species and stage are visualized in isobolograms in (Fig. S1). The associated FIC_A/B_ values are summarized in (Tables S3 and S4).

**TABLE 1:** FIC index values for emodepside in combination with partner drugs at three fixed ratios (3:1, 2:2, 1:3 emodepside:partner drug [v:v]) against larval stages of different STH models^*b*^^,^^*c*^

Partner drugs	*H. polygyrus*			*N. americanus*			*A. ceylanicum*			*T. muris*			*S. ratti*		
	3:1	2:2	1:3	3:1	2:2	1:3	3:1	2:2	1:3	3:1	2:2	1:3	3:1	2:2	1:3
Abamectin	0.06	0.08	0.11	1.70	1.71	0.81	1.36	1.73	1.91	*^a^			0.80	0.83	0.58
Ivermectin	0.58	0.39	0.56	1.72	0.76	1.69	1.18	0.78	0.89	*^a^			1.18	1.22	1.84
Moxidectin	0.54	0.69	2.52	0.88	1.01	1.73	1.49	1.15	0.65	*^a^			1.01	2.19	3.80
Levamisole	1.58	1.49	1.79	*^a^			*^a^			*^a^			1.17	1.45	3.68
Oxantel pamoate	*^a^			*^a^			*^a^			0.76	0.33	0.64	*^a^		
Pyrantel pamoate	0.90	1.11	0.54	*^a^			*^a^			1.09	0.86	1.34	0.85	1.04	1.19
Tribendimidine	*^a^			*^a^			*^a^			*^a^			1.23	1.52	0.96

^*a*^ *see Fig. 2; Fig. S2, and Table S5.

^*b*^ FIC < 0.9 synergy, FIC = 0.9–1.5 additivity, FIC > 1.5 antagonism.

^*c*^ All FIC values were calculated based on EC_50_ values determined 72 h post-incubation.

**TABLE 2:** FIC index values for emodepside in combination with partner drugs at three fixed ratios (3:1, 2:2, 1:3 emodepside:partner drug [v:v])^*a*^^,^^*d*^^,^^*e*^

Partner drugs	*H. polygyrus*			*T. muris*			*S. ratti*			
	3:1	2:2	1:3	3:1	2:2	1:3	3:1	2:2	1:3	
Albendazole	*^b^			0.94	0.77	0.59	n.a.			
Mebendazole	*^b^			0.68	1.12	1.35	n.a.			
Albendazole sulfoxide^*c*^	*^b^			0.29	0.58	0.32	n.a.			
Abamectin	n.a.			n.a.			*^b^			
Ivermectin	*^b^			0.91	0.37	0.9	1.18	1.46		1.75
Moxidectin^*c*^	*^b^			0.33	0.37	0.57	0.69	0.99		0.69
Levamisole^*c*^	1.25	0.88	1.02	0.48	1.28	1.18	n.a.			
Oxantel pamoate	*^b^			0.94	0.86	1.1	n.a.			
Pyrantel pamoate	1.80	1.49	0.59	0.55	0.55	0.63	n.a.			
Tribendimidine	3.23	1.14	1.19	0.83	0.88	1.46	n.a.			

^*a*^ n.a.: not applicable.

^*b*^ *see Fig. 3, Fig. S3, and Table S7.

^*c*^ The FIC values were determined from one experiment only.

^*d*^ All FIC values were calculated based on EC_50_ values determined 72 h post-incubation.

^*e*^ FIC < 0.9 synergy, FIC = 0.9-1.5 additivity, FIC > 1.5 antagonism.

Against adult *T. muris,* the combination of emodepside and albendazole or albendazole sulfoxide showed mostly synergism, with ΣFIC values between 0.29 and 0.94, with no ratio-dependent pattern. The combination emodepside-mebendazole resulted in synergism only at a ratio of 3:1 (ΣFIC = 0.68), while ratios of 2:2 and 1:3 showed additivity (ΣFIC = 1.12 and 1.35, respectively).

Combinations of emodepside with macrocyclic lactones showed species-dependent interactions. Against *H. polygyrus* L3, all combinations and ratios exhibited synergism with ΣFIC values between 0.06 and 0.69. The only exception was emodepside-moxidectin at 1:3 (ΣFIC = 2.52), which showed antagonism. In contrast, only a few drug combinations and ratios showed synergistic responses against the other two hookworm species. Against *N. americanus* L3, only one ratio per combination showed synergy (emodepside-moxidectin 2:2, ΣFIC = 0.88; emodepside-ivermectin 3:1, ΣFIC = 0.76; and emodepside-abamectin 1:3, ΣFIC = 0.81). However, against *A. ceylanicum* L3, emodepside-ivermectin yielded two synergistic ratios (2:2, ΣFIC = 0.78 and 1:3, ΣFIC = 0.89), and emodepside-moxidectin only one synergistic ratio (1:3, ΣFIC = 0.65). Against *S. ratti* L3, only the combination emodepside-abamectin showed synergy across all tested ratios (ΣFIC = 0.58–0.83),while the combination between emodepside-ivermectin showed a trend toward antagonism (ΣFIC = 1.18–1.84). Against adult *T. muris*, the combination emodepside-ivermectin or moxidectin showed mostly synergism, with ΣFIC values ranging from 0.33 to 0.91. For the combination with moxidectin, we observed ratio-dependent synergism, with the ratio 3:1 (ΣFIC = 0.33) showing the greatest synergy. This effect was not observed for the combination with ivermectin. Overall, the combination emodepside-moxidectin demonstrated greater synergistic potential than that with ivermectin. In contrast, against adult *S. ratti,* the emodepside-ivermectin combination showed a trend toward antagonism (ΣFIC = 1.18–1.75), increasing in magnitude with higher emodepside proportions and suggesting a ratio-dependent pattern. On the other hand, the combination with moxidectin showed synergy at the 3:1 and 1:3 ratios (ΣFIC = 0.69 each) and additivity at the 2:2 ratio (ΣFIC = 0.99), without a consistent ratio-dependent pattern. Due to the limited activity of macrocyclic lactones against adult *H. polygyrus*, FIC analysis was not possible for these combinations.

The combinations of emodepside and different nAChR agonists showed species- and stage-specific interaction patterns. Against *H. polygyrus* L3, emodepside-pyrantel pamoate showed additive effects at ratios 3:1 (ΣFIC = 0.9) and 2:2 (ΣFIC = 1.11), and synergy at ratio 1:3 (ΣFIC = 0.53). The combination emodepside-levamisole showed antagonistic effects across all ratios (ΣFIC = 1.49–1.79). On adult *H. polygyrus,* this combination showed a shift toward additivity (ΣFIC = 0.88–1.25). The combination of emodepside-pyrantel pamoate showed a ratio-dependent pattern, shifting from antagonism to synergy (ΣFIC = 1.80–0.59). In comparison, emodepside-tribendimidine shifted from additivity toward antagonism (ΣFIC = 1.14–3.23).

Against *T. muris* L1, emodepside-oxantel pamoate exhibited synergy across all ratios (ΣFIC = 0.33–0.64), while emodepside-pyrantel pamoate showed additivity across almost all ratios (ΣFIC = 0.86–1.34). In contrast to the larval stage, emodepside-pyrantel pamoate was synergistic against adult *T. muris* at all ratios, with ΣFIC = 0.55–0.63. In comparison, the combination emodepside-oxantel pamoate was not as potent, shifting toward additivity (ΣFIC = 0.86–1.1). For the emodepside-levamisole combination, only the 3:1 ratio was synergistic (ΣFIC = 0.48), while the 2:2 and 1:3 ratios were additive (ΣFIC = 1.28 and 1.18, respectively). The emodepside-tribendimidine combination exhibited a ratio-dependent potency, shifting from synergy toward additivity (ΣFIC = 0.83–1.46) with decreasing emodepside concentrations. Similar to the other larval species, *S. ratti* L3 showed additive, synergistic, or antagonistic responses to emodepside combinations with nAChR agonists. For example, emodepside-levamisole and emodepside-tribendimidine demonstrated a shift toward antagonism (ΣFIC = 1.17–3.68). In contrast, the emodepside-pyrantel pamoate combination showed a shift from synergy to additivity (ΣFIC = 0.85–1.19).

Overall, no consistent ratio-dependent pattern was observed, with the optimal ratio varying by drug, species, and life stage.

### Non-ratio drug combinations

**Fig 2:**
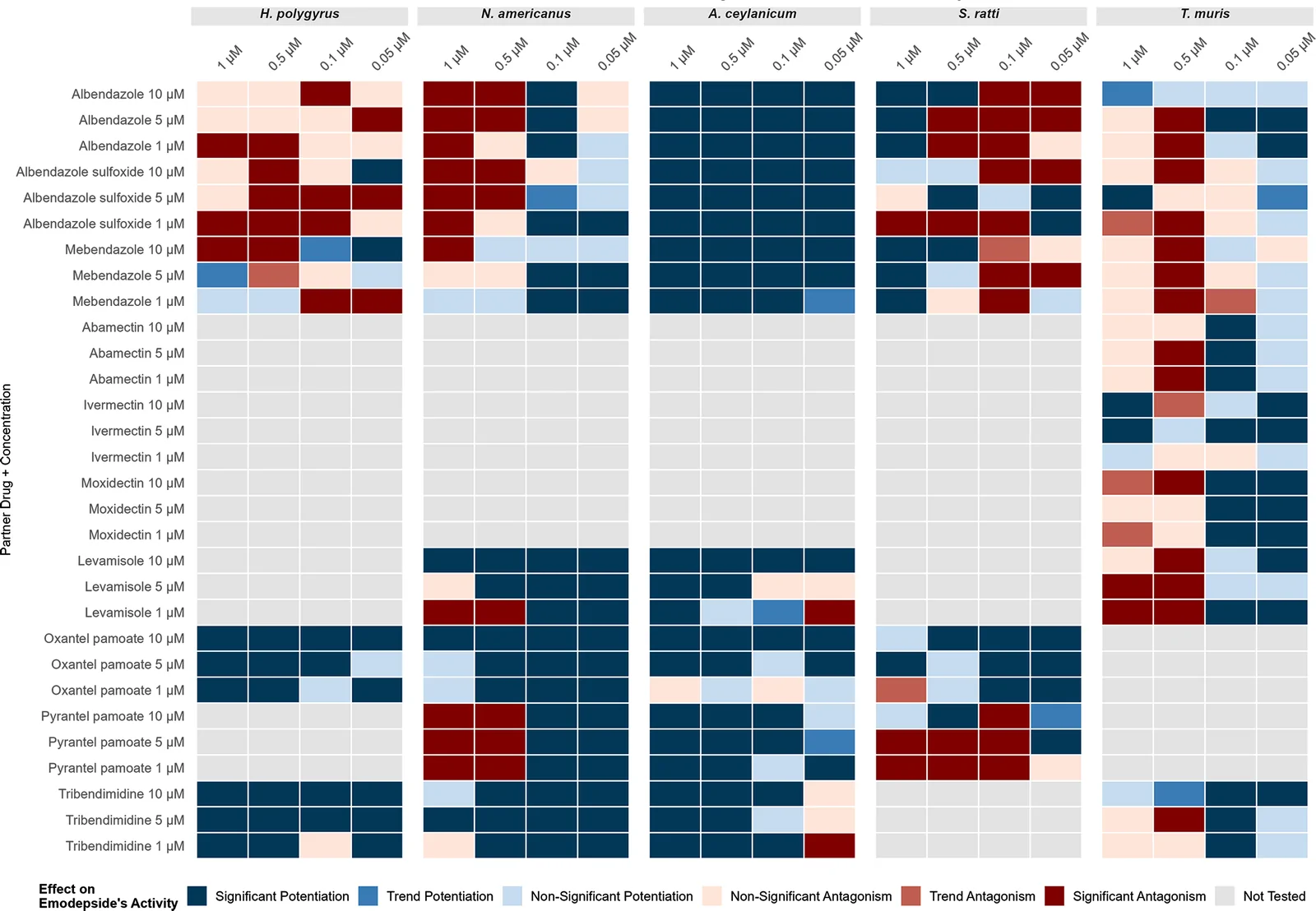
Effect of partner drugs on emodepside’s activity on the larval stage. The heatmap displays the interaction effect of anthelmintic partner drugs at three fixed concentrations (1, 5, and 10 μM) in combination with four fixed concentrations of emodepside (0.05, 0.1, 0.5, and 1 μM) on STH larval stages. Interactions were classified based on GLM analysis. Gray cells indicate conditions that were not tested with this method.

**Fig 3:**
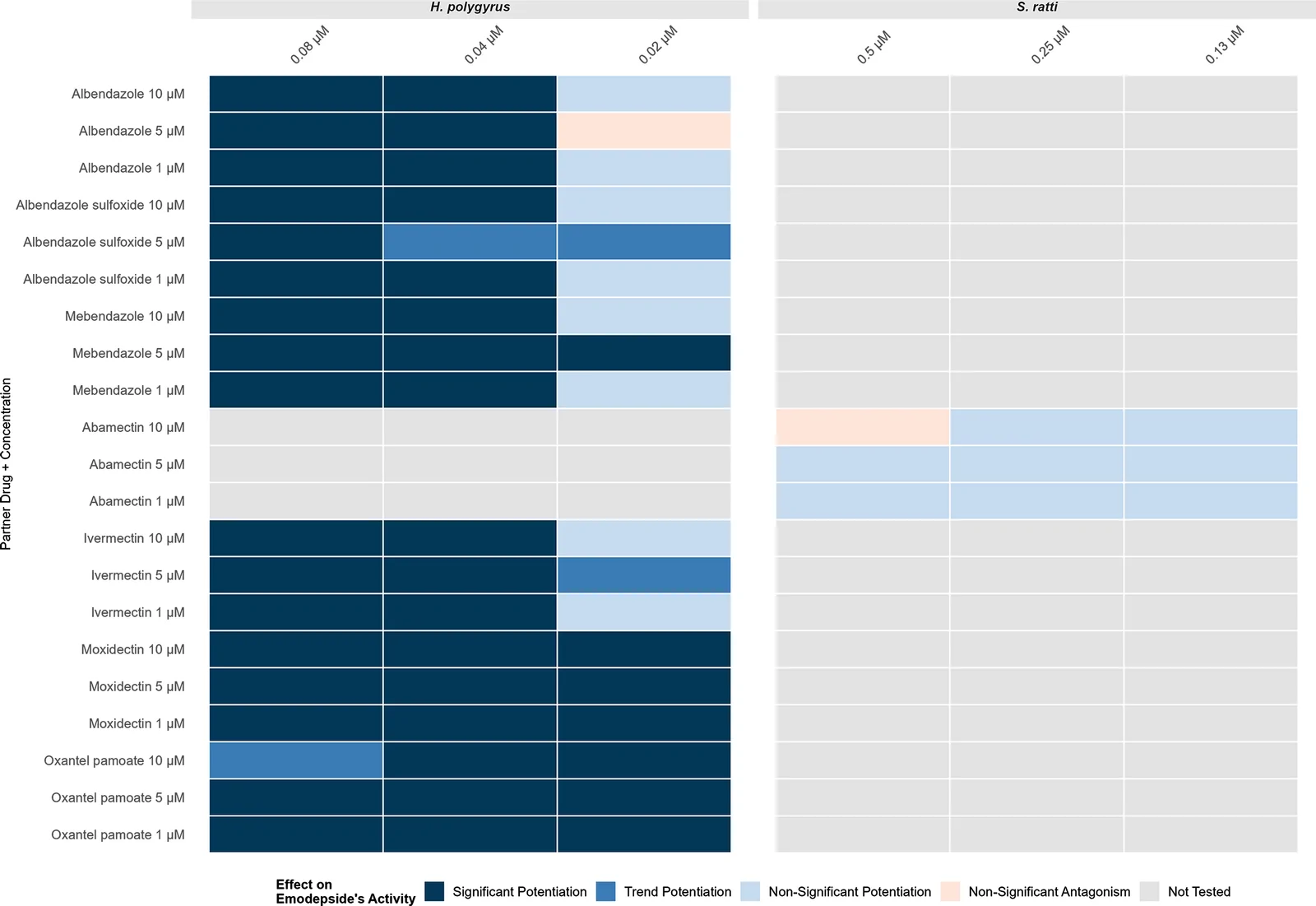
Effect of partner drugs on emodepside’s activity on STH adult stages. The heatmap displays the interaction effect of anthelmintic partner drugs at three fixed concentrations (1, 5, and 10 μM) in combination with three fixed concentrations of emodepside (0.08, 0.04, and 0.0*2* μM *H. polygyrus* and 0.5, 0.25, and 0.13 μM *S. ratti*) on STH adult stages. Interactions were classified based on a linear mixed-effects regression model (LMER) analysis. Gray cells indicate conditions that were not tested with this method.

To assess the combined effect of partner drugs on the activity of emodepside, predicted absolute survival percentages for individual emodepside concentrations were modeled using a GLM, comparing single-drug emodepside to combination treatments (Fig. S2). Overall, the effect of partner drugs on emodepside’s activity varied by drug class, species, life stage, and concentration. Based on scatterplot analysis (not shown), the emodepside concentration range of 0.05 µM–1 µM showed the greatest variability between single and combination treatments in larvae; the pairwise difference between the two groups across this range is visualized as a heatmap (Fig. 2). In adult worms, the relevant LMER concentration range was determined individually per species: *H. polygyrus* (0.08 µM–0.02 µM) and *S. ratti* (0.5 µM–0.1 µM), with the corresponding heatmap presented in Fig. 3.

On *H. polygyrus* L3, the benzimidazoles only marginally altered emodepside’s activity (EC_50_ range: 1.1 µM–1.9 µM) (Fig. 2; Table S5, Fig. S2). The combination of albendazole sulfoxide and emodepside showed a reverse concentration-dependent antagonistic pattern (10 µM EC_50_ = 1.5 ± 0.2 µM; 5 µM EC_50_ = 1.7 ± 0.2 µM; 1 µM EC_50_ = 1.9 ± 0.4 µM). Mebendazole (10 µM) showed a dual effect: potentiation at lower emodepside concentrations (0.01 µM–0.1 µM) and antagonism at higher concentrations (5 µM–50 µM). In contrast, in adult *H. polygyrus* worms, the combination of emodepside with the three benzimidazoles produced heterogeneous interaction profiles. All three drugs showed significant potentiation of emodepside’s activity at the two higher emodepside concentrations (0.08 and 0.04 µM). Below 0.02 µM, potentiation was no longer significant for most combinations, except for albendazole and albendazole sulfoxide at 5 µM, which retained significant-to-trend potentiation (Fig. 3). The corresponding dose-response curves (Fig. S3) confirmed these patterns, with the combination groups displaying a leftward shift relative to the emodepside-alone control, most pronounced at the higher benzimidazole concentrations, indicating an overall potentiating effect across all three benzimidazoles. The calculated EC_50_ values were consistently in the range of 0.03 µM–0.04 µM, representing an approximately threefold potentiation compared to emodepside alone (Table S7). Against *A. ceylanicum* L3, all three benzimidazoles potentiated emodepside’s activity in a concentration-dependent manner (Fig. 2; Table S5, Fig. S2). Albendazole sulfoxide showed the most pronounced potentiation, reducing emodepside’s EC_50_ from 2.0 ± 0.5 µM to 0.1 µM at both 10 and 5 µM, resulting in a 20-fold decrease, and to 0.3 ± 0.3 µM at 1 µM (Table S5). Unlike against *A. ceylanicum* L3, the benzimidazoles predominantly antagonized emodepside against *N. americanus* L3. Albendazole sulfoxide (10, 5, and 1 µM) and albendazole (10 µM) caused the largest right-side EC_50_ shift, ranging from 2.5- to 16-fold (Fig. 2; Table S5, Fig. S2). Mebendazole and albendazole at lower concentrations (5 and 1 µM) did not considerably alter emodepside’s activity. However, a concentration-dependent behavior was apparent, resulting in potentiation at lower emodepside concentrations (0.05 and 0.1 µM) and antagonism at higher concentrations (0.5 and 1 µM) (Fig. 2).

The benzimidazoles had only minor effects on emodepside’s activity on *H. polygyrus* L3, *S. ratti* L3 and *T. muris* L1 (Fig. 2; Table S5, Fig. S2). Against *S. ratti* L3, pairwise GLM comparison revealed significant antagonism specifically at lower emodepside concentrations (0.05 and 0.1 µM) in the albendazole and mebendazole combinations, while higher concentrations trended toward potentiation. In contrast, against *T. muris* L1, pairwise GLM comparisons were scattered across concentrations without a consistent directional pattern (Fig. 2). Notably, the lowest tested concentration (1 µM) consistently produced the largest EC_50_ shifts across all three drugs, moving emodepside’s EC_50_ from 0.3 ± 0.01 µM to 0.6 ± 0.06 µM (albendazole), 0.8 ± 0.02 µM (mebendazole), and 0.7 ± 0.2 µM (albendazole sulfoxide), showing an inverse concentration-response relationship.

Similar to benzimidazoles, macrocyclic lactones only marginally affected emodepside’s activity (Table S5, Fig. S2) on *T. muris* L1; however, pairwise GLM comparison showed a significant difference toward potentiation at lower emodepside concentrations (0.05 µM–0.1 µM) (Fig. 2). Against adult *H. polygyrus,* the combination of emodepside with ivermectin and moxidectin showed consistent statistically significant potentiation across the tested emodepside concentrations. Both macrocyclic lactones reduced the EC_50_ of emodepside into the 0.03–0.05 range compared to emodepside alone (EC_50_ = 0.1 ± 0.01 µM). In contrast, against adult *S. ratti*, the combination of emodepside with abamectin did not show any potentiated effect, as the EC_50_ of emodepside in the presence of different abamectin concentrations (0.2 µM–0.3 µM) remained comparable to that of emodepside alone (Fig. 3; Table S7, Fig. S3).

Against H. *polygyrus* L3, oxantel pamoate and tribendimidine, and against *A. ceylanicum* L3, levamisole, oxantel pamoate, pyrantel pamoate, and tribendimidine potentiated emodepside in a concentration-dependent manner (Table S5, Fig. S2). Consistent with the effect observed against *H. polygyrus* L3, against adult worms, all tested oxantel pamoate concentrations (1, 5, and 10 µM) reduced the EC_50_ of emodepside, with pairwise LMER comparison confirming statistically significant potentiation at almost all concentrations (Fig. 3; Fig. S3). Overall, this combination resulted in up to fivefold potentiation of emodepside’s efficacy. On *N. americanus* L3, levamisole, oxantel pamoate, pyrantel pamoate, and tribendimidine showed divergent effects on emodepside’s activity. Levamisole showed the strongest potentiation at 0.01 µM emodepside, revealing a reduction of predicted survival from ~95% with emodepside alone to 44%–59%, in a concentration-dependent manner (Fig. 2; Fig. S2). Accordingly, EC_50_ values decreased concentration-dependently with increasing levamisole concentrations, with the two highest concentrations of emodepside (10 and 5 µM) resulting in a 40- and 10-fold decrease, respectively (Table S5). Oxantel pamoate showed an unusual reverse concentration-dependent potentiation in survival curves, though EC_50_ values followed a conventional concentration-dependent decrease, and pairwise GLM confirmed overall significant potentiation (Fig. 2; Table S5, Fig. S2). Pyrantel pamoate showed a dual pattern, with significant antagonism at higher emodepside concentrations (0.5 and 1 µM) and potentiation at lower concentrations (0.05 and 0.1 µM), with EC_50_ values confirming a rightward shift consistent with overall antagonism (Fig. 2; Table S5). Against *S. ratti* L3, oxantel pamoate and pyrantel pamoate had opposing effects. Oxantel pamoate potentiated emodepside concentration-dependently, while pyrantel pamoate showed significant antagonism in the pairwise GLM comparison despite only marginal EC_50_ shifts (Fig. 2; Table S5, Fig. S2). Against *T. muris* L1, levamisole showed a marginal reverse concentration-dependent influence on emodepside’s EC_50_ (Table S5), while the pairwise GLM comparison revealed a significant antagonistic difference at lower concentrations of emodepside (0.05 and 0.1 µM) (Fig. 2). A lower levamisole concentration (1 µM) exerted greater influence on emodepside’s activity than the highest tested concentration (10 µM), with EC_50_ values of 1.0 ± 0.3 and 0.5 ± 0.03 µM, respectively (Table S5). Tribendimidine produced significant potentiation at lower emodepside concentrations (0.1 µM across all tribendimidine concentrations; 0.05 µM at 10 µM tribendimidine), though EC_50_ values remained only marginally different from emodepside alone (Fig. 2; Table S5, Fig. S2). Consistent with the finding against adult *H. polygyrus,* oxantel pamoate also significantly potentiated emodepside’s activity, as confirmed by a reduction of emodepside’s EC_50_ from 0.01 µM to 0.002 µM, reflecting an approximately fivefold potentiation (Fig. 3; Table S7, Fig. S3).

### CYP450-based DDI evaluation

All calculated IC_50_ values for the individual drugs and combinations tested on selected CYP450 enzymes are summarized in Table 3. The statistical evaluation of the pairwise comparison is summarized in Fig. S4.

**TABLE 3:** Summarized IC₅₀ (μM ± [SE]) values of selected anthelmintics and emodepside-based combinations against major hepatic CYP450 enzymes^*a*^^,^^*b*^^,^^*c*^

Single drugs	CYP 1A2	CYP 2C9	CYP 2C19	CYP 2D6	CYP 3A4
Emodepside	>100	37.2 [11.6]	>100	>100	4.2 [0.7]
Levamisole	>100	>100	>100	24.0 [4.3]	>100
Oxantel pamoate	>100	15.0 [4.9]	<100^*a*^	10.7 [1.4]	62.7 [26.9]
Pyrantel pamoate	<100	<100	>100	<100	>100
Tribendimidine	>100^*a*^	>100	>100	<100	>100
Albendazole	83.5 [20.6]	>100	>100	>100	>100
Mebendazole	12.5 [1.4]	24.5 [2.9]	>100	>100	>100
Albendazole sulfoxide	<100	>100	>100	>100	>100
Ivermectin	>100	6.7 [0.6]	>100	24.4 [6.1]	12.1 [3.4]
Moxidectin	>100	<100	>100	<100	>100
Combination					
Emodepside-levamisole	>100	60.8 [21.1]	>100	13.1 [2.3]	8.5 [3.3]
Emodepside-oxantel pamoate	82.6 [12.8]	8.4 [0.9]	50.4 [36.0]	9.8 [1.6]	19.8 [11.0]
Emodepside-pyrantel pamoate	41.9 [3.5]	10.7 [0.9]	83.8 [47.4]	4.5 [0.4]	10.9 [7.3]
Emodepside-tribendimidine	>100	>100	>100	>100	7.6 [2.1]
Emodepside-albendazole	>100	>100	>100	>100	6.0 [2.4]
Emodepside-mebendazole	>100	>100	>100	>100	6.2 [4.3]
Emodepside-albendazole sulfoxide	17.0 [2.6]	>100	>100	>100	6.9 [3.6]
Emodepside-ivermectin	>100	7.2 [1.0]	>100	29.8 [11.4]	8.1 [2.5]
Emodepside-moxidectin	>100	3.1 [0.5]	>100	36.5 [9.5]	4.8 [1.3]

^*a*^ Tribendimidine was tested in a single experimental replicate only.

^*b*^ IC₅₀ could not be determined due to insufficient data points in the upper part of the sigmoidal curve, preventing reliable curve fitting.

^*c*^ SE: standard error.

#### CYP1A2

Ivermectin, moxidectin, emodepside, levamisole, oxantel pamoate, and tribendimidine showed no meaningful inhibition of CYP1A2 (IC_50_ >100 µM). Pyrantel pamoate inhibited CYP1A2 > 60% (Table S8) at the highest concentration tested (100 μM). Among the benzimidazoles, albendazole showed weak inhibition (IC_50_ = 83.5 ± 20.6 µM), while albendazole sulfoxide showed higher inhibitory activity, inhibiting >80% of the CYP1A2 at 100 μM. Mebendazole demonstrated the lowest IC_50_ value (12.5 ± 1.4 µM).

The emodepside-oxantel pamoate and emodepside-pyrantel pamoate combinations show weak inhibition (IC_50_ = 82.6 ± 12.8 µM and 41.9 ± 3.5 µM, respectively). The emodepside-mebendazole combination eliminated the inhibition observed with mebendazole alone, with the IC_50_ shifting from 12.5 μM to >100 µM (≥8-fold increase). Contrarily, emodepside-albendazole sulfoxide developed weak inhibitory activity (IC_50_ = 17.0 ± 2.6 µM) despite neither individual drug showing inhibition.

#### CYP2C9

Albendazole, albendazole sulfoxide, levamisole, and tribendimidine showed no inhibitory effect on CYP2C9. Weak inhibition was observed for emodepside (IC_50_ = 37.2 ± 11.6 µM), mebendazole (IC_50_ = 24.5 ± 2.9 µM), and oxantel pamoate (IC_50_ = 17.0 ± 2.6 µM). Ivermectin exhibited the strongest inhibition among individually tested drugs (IC_50_ = 6.7 ± 0.6 µM), while pyrantel pamoate and moxidectin, tested only at 100 μM, inhibited CYP2C9 by 75% and 100%, respectively (Table S8).

Consistent with the lack of inhibition by individual drugs, combinations of emodepside and albendazole, albendazole sulfoxide, or tribendimidine showed no inhibition. The emodepside-mebendazole combination lost inhibitory activity (IC_50_ > 100 µM), despite both drugs showing weak inhibition individually. The most pronounced IC_50_ shift was seen with emodepside-moxidectin (IC_50_ = 3.1 ± 0.5 µM, 12-fold decrease), followed by emodepside-ivermectin (IC_50_ = 7.2 ± 1.0 µM, 5-fold decrease), and emodepside-oxantel pamoate and (IC_50_ = 8.4 ± 0.9 µM, 4-fold decrease). The combination emodepside-pyrantel pamoate elicited the lowest inhibitory potential (IC_50_ = 10.65 ± 0.9 µM, 4-fold decrease).

#### CYP2C19

No individual drug produced a calculable IC_50_ for CYP2C19. Oxantel pamoate inhibited the enzyme >80% at 100 μM but without a complete dose-response curve, and all other drugs showed no inhibition. Most of the combinations were similarly inactive (IC_50_ > 100 µM). Only two combinations developed weak inhibitory activity: emodepside-pyrantel pamoate (IC**_50_** = 83.8 ± 47.4 µM) and emodepside-oxantel pamoate (IC_50_ = 50.4 ± 36.0 µM).

#### CYP2D6

The benzimidazoles and emodepside showed no inhibitory effects (IC_50_ > 100 µM). Weak inhibition was observed for ivermectin (IC_50_ = 24.4 ± 6.1 µM), levamisole (IC_50_ = 24.0 ± 4.3 µM), and oxantel pamoate (IC_50_ = 10.7 ± 1.4 µM). Pyrantel pamoate, moxidectin, and tribendimidine, each tested at 100 μM, inhibited CYP2D6 by 93.7 ± 0.4 %, 72.3 ± 10.6%, and 52.6 ± 0.2%, respectively (Table S8).

Consistent with the individual drugs, combinations with benzimidazoles and tribendimidine showed no inhibitory effects. Emodepside-moxidectin (IC_50_ = 36.5 ± 9.5 µM) and emodepside-ivermectin (IC_50_ = 29.8 ± 11.4 µM) demonstrated low inhibitory effects, similar to the individual drugs. The emodepside-levamisole combination (IC_50_ = 13.1 ± 2.3 µM) showed a 1.8-fold increase in inhibitory effect, compared to levamisole alone. Emodepside-oxantel pamoate (IC_50_ = 9.8 ± 1.6 µM) and emodepside-pyrantel (IC50 = 4.5 ± 0.4 µM) exhibited the strongest yet moderate inhibition on CYP2D6.

#### CYP3A4

Oxantel pamoate (IC_50_ = 62.7 ± 26.9 µM) and ivermectin (IC_50_ = 12.1 ± 3.4 µM) showed weak inhibition, while emodepside exhibited the strongest yet moderate inhibition of all tested drugs (IC_50_ = 4.2 ± 0.7 µM). All other tested drugs showed no inhibitory effect on CYP3A4 (IC_50_ > 100 µM).

None of the emodepside combinations exceeded the potency of emodepside alone. The emodepside-oxantel pamoate combination showed the weakest inhibition (IC_50_ = 19.8 ± 11.0 µM), representing a 4.7-fold reduction in potency compared to emodepside alone. Combinations with empodepside and nAChR agonists or ivermectin ranged from 8.1 to 10.9 μM, reflecting a 1.9- to 2.6-fold reduction in potency to emodepside alone. The three benzimidazoles’ combinations showed similar inhibitory potencies, with IC_50_ values around 6.0 μM. The emodepside-moxidectin combination showed the most potent inhibition among all combinations (IC_50_ = 4.8 ± 1.3 µM), only marginally different from emodepside alone

## DISCUSSION

The current control of STH infections relies on two benzimidazoles, albendazole and mebendazole, both clinically unsatisfactory and showing variable efficacy, especially against *T. trichiura* and hookworm. For *S. stercoralis*, ivermectin remains the recommended treatment, though its use is contraindicated in *Loa loa*-co-endemic areas, and it is used in a weight-dependent manner, which complicates further the distribution in MDA settings. Against this background, emodepside emerged as a promising repurposed candidate. Combining emodepside with existing anthelmintics could further enhance its efficacy, particularly against hookworms. The potential for enhancing emodepside’s efficacy through combination has been previously demonstrated by co-incubation of adult *Brugia malayi* with diethylcarbamazine (39). An emodepside combination could also delay the decrease of efficacy, which has been reported for the two widely distributed benzimidazoles, albendazole and mebendazole (7), or resistance evolution. This is a particularly relevant consideration given that STH control programs rely on large-scale distribution of the drugs. As combination regimens introduce additional complexity of potential pharmacokinetic interactions, a systematic evaluation of both combined anthelmintic activity and CYP450-mediated DDI risk is needed. CYP isozymes metabolize approximately 80% of the drugs currently available on the market (40), and when co-administered drugs share the same metabolic enzyme, inductive or inhibitory effects can mutually alter plasma drug levels with consequences for both efficacy and safety (41). From the literature, albendazole has been reported to induce dose-dependently CYP1A2 (42), while no clinically relevant induction has been reported for the remaining partner compounds tested in this study. In the present work, we evaluated emodepside in combination with a range of partner drugs against multiple STH species and life stages *in vitro* and assessed the inhibitory potential of these combinations on major hepatic CYP450 enzymes.

### Emodepside—a suitable combination partner

The present study confirms the previously observed broad-spectrum activity of emodepside reported in preclinical and clinical studies (22, 24, 25). This broad-spectrum potency was not observed for the benzimidazoles, which did not exhibit activity (EC_50_ > 100 µM) at the larval stage against any tested species and only limited activity against adult worms, while the macrocyclic lactones and drugs active on the nAChR demonstrated species- and stage-dependent potency. The findings were in accordance with previously reported studies (30).

Despite the lack of activity in the motility-based assay, benzimidazoles elicited diverse effects on emodepside’s activity, in a species- and stage-dependent manner. Among all tested larval species, *A. ceylanicum* was the only species to show potentiation, with reduction of emodepside’s EC_50_ up to 20-fold, while the closely related *N. americanus* (43) and all other tested larval species showed no effect on emodepside’s EC_50_. These contrasting results may reflect biological differences between the two hookworm species. *A. ceylanicum* larvae have been shown to be inherently more sensitive to anthelmintic compounds than *N. americanus* across drug classes *in vitro* (43); hence, possibly, this species is more susceptible to combination-induced effects even when individual drugs show no measurable single-drug activity.

In contrast to the larval data, emodepside combination with all three benzimidazoles produced consistent potentiation against adult worms of *H. polygyrus,* and emodepside combined with albendazole and albendazole sulfoxide resulted in mostly synergistic ratios against *T. muris*, suggesting that developmental stage may be a primary determinant of whether the combination chemotherapy exhibits antiparasitic activity rather than species for this class. This pattern aligns with the documented greater susceptibility of adult worms to benzimidazoles compared to larvae (30, 44). Both albendazole and mebendazole interact with the same drug target, the disruption of β-tubulin microtubules. *In vitro*, both drugs show similar activity against adult *T. muris*. We therefore cannot explain the differences in the interaction observed between emodepside-albendazole/albendazole sulfoxide (ΣFIC 0.29–0.94, synergistic trend) and emodepside-mebendazole (ΣFIC 0.68–1.35, additive trend).

We observed that the combination between emodepside and macrocyclic lactones was especially favorable against *H. polygyrus* larvae (ΣFIC 0.06–2.52), across most of the tested ratios. In contrast, the other larval species tested exhibited variable combination effects with no clear pattern. We cannot explain the observed variability as both drugs lead to the same endpoint: flaccid paralysis. Macrocyclic lactones act by opening the glutamate-gated chloride channels (GluCls), producing a hyperpolarizing effect on the nematode’s neuromuscular function and producing a sustained increase in Cl⁻ conductance (18, 45). On the other hand, emodepside hyperpolarizes the cell membrane by activating the calcium- and voltage-gated SLO-1 (BK) potassium channel and increasing voltage-activated K^+^ current (45). When co-activating both channels, thus increasing the SLO-1 K^+^ and Cl⁻ conductance, one would expect reinforced neuromuscular inhibition and, hence, additive or synergistic behavior.

Tested combination against adult worms showed very variable responses. The combination consistently potentiated emodepside’s EC_50_ values against *H. polygyrus* and produced synergistic ratios in *T. muris*, whereas in *S. ratti* the effects of the combination were dependent on the macrocyclic lactone tested. The enhancement effect might be explained by both macrocyclic lactones and emodepside being P-glycoprotein transporter (Pgp) substrates (46, 47). Molecular modeling of *Caenorhabditis elegans* Pgp-1 (Cel-Pgp-1) revealed that macrocyclic lactones and emodepside have an overlapping binding site, suggesting that in combination the molecules could compete for the same efflux pump and result in an accumulation inside the cell (48). The diversity of the effect might be explained by species-specific differences in Pgp gene number and expression levels, as nematode species can considerably differ in both (49). Also, individual macrocyclic lactones have been shown to differ in their interaction with the nematode Pgp and in their effects on GluCl subunits (46, 50). Identifying which determinant is responsible would require species- and compound-resolved transport and receptor studies.

Combinations involving drugs targeting the nAChRs showed a heterogeneous pattern across species and life stages. The heterogeneity might be dependent on the receptor subtype that these drugs target and likely correlates to the relative abundance and functional contribution of these receptor subtypes across species and life stages (51, 52). nAChRs consist of multiple protein subunits; the combination and ratio of these determine which drugs the receptor responds to. It has been demonstrated that altering the ratio of expressed subunits is sufficient to shift the pharmacological profile of the receptor, reproducing distinct drug-sensitivity subtypes. Consequently, closely related species may respond very differently to the same drug combination if they differ in their relative subunit expression, thus effectively possessing functionally distinct receptors despite sharing the same underlying subunit genes (53). The differences in susceptibility from larval to adult stage, as observed for *H. polygyrus* and *T. muris* for the combination emodepside-oxantel pamoate, may reflect a similar principle. The proportion of different nAChR types present can depend on the developmental stage, thereby altering its sensitivity to drug combinations.

### Absence of relevant CYP450 inhibition in combination *in vitro* studies

Our study confirms that emodepside inhibits CYP3A4 (IC₅₀ = 4.2 ± 0.7 µM); however, it is not at clinically relevant exposure (54). Changes in the exposure of emodepside in the presence of co-medication acting either as a potent CYP3A4 inhibitor or inducer cannot be ruled out and hence require further assessment based on overall *in vitro* and *in vivo* profiles. Reassuringly, in this study, none of the combinations with emodepside showed stronger inhibition of CYP3A4 than emodepside alone, and none of the tested combinations showed potent inhibition potential (<1 µM) (54). Across all other CYP isoforms, the combinations’ inhibitory signals were most plausibly driven by the individual compounds/drugs rather than increased inhibitory effects. The only exception was the emodepside-albendazole sulfoxide combination (17.0 [2.6] μM), which is consistent with the intrinsic CYP1A2 inhibitory activity previously reported for albendazole sulfoxide (14.0 μM) (41), also suggesting the signal is referable to the partner drug.

Among the partner drugs, ivermectin’s inhibition profile toward CYP2D6, CYP3A4, and CYP2C9 was consistent with the literature, though the absence of CYP2C19 inhibition did not align with some of the earlier reports, highlighting that CYP inhibition profiles of anthelmintics can be sensitive to experimental conditions (55). Moxidectin inhibited CYP2C9 and CYP2D6 at 100 μM, displaying a pattern consistent with the structurally related ivermectin and suggesting a potential class effect within the macrocyclic lactone scaffold (55). Within the benzimidazole class, mebendazole showed inhibitory activity broadly consistent with prior reports, whereas albendazole and albendazole sulfoxide exhibited low to no inhibition of CYP1A2 (41). Oxantel pamoate’s inhibition profile was less pronounced than in previous studies, while pyrantel pamoate substantially inhibited CYP1A2, CYP2C9, and CYP2D6 at 100 μM. Given the poor systemic absorption of both compounds, these findings are unlikely to be of clinical relevance (41, 56, 57). Levamisole and tribendimidine showed negligible to weak CYP inhibition, with clinically irrelevant DDI liability expected under therapeutic conditions (54, 56). Statistical analysis of the pairwise comparison revealed that, in most cases, the CYP inhibition signal for the tested combinations differed significantly from that of the single drugs at the three highest concentrations tested. Additionally, statistical evaluation of both single drugs and their combination showed that the combination differed significantly from one of its two partner drugs but never from both. Overall, the measured IC_50_ values for tested drugs and combinations substantially exceeded plasma levels expected under therapeutic dosing; the clinical relevance of the observed CYP inhibition signals is considered low.

### Limitations

This study has several limitations. First, motility-based readouts have inherent constraints and cannot fully capture drug efficacy. Not all anthelmintic drug classes act through mechanisms that produce an observable effect on motility, meaning that the absence of immobility does not necessarily indicate the absence of drug activity against the parasite. This is particularly relevant for benzimidazoles, which show low to no activity in motility-based assays. For example, albendazole can also act as a spindle apparatus disruptor, arresting mitosis at the G2/M phase and triggering cell death (58). Consistent with this mechanism, it showed activity by preventing *H. polygyrus* eggs from hatching (59).

Second, we did not quantify the actual drug concentrations present in the assay media. Most compounds tested are practically insoluble in water, and the concentration effectively available to the larval and adult stages may therefore be lower than intended. During the assay preparation, compounds were transferred from DMSO stock dilution into aqueous-based media. Although no visible precipitation was observed for compounds across the lower concentration range (<50 µM), we cannot exclude sub-visible colloidal aggregation. However, concentration-dependent responses across the tested range suggest that the amount of dissolved drug increased gradually, rather than reaching its solubility limit. Nevertheless, the reported concentrations should be interpreted with caution. The absence of an effect at high concentrations may reflect lower-than-intended drug exposure rather than a true lack of drug activity. Future studies would benefit from quantifying free drug concentrations in the assay media by liquid chromatography-tandem mass spectrometry (LC-MS/MS) to confirm the effective doses and to distinguish genuine pharmacological activity from potential aggregation-related artifacts.

### Conclusion

In conclusion, the present study provides a thorough evaluation of emodepside as a combination partner against STHs *in vitro*, representing an important step toward the development of novel combination therapies. Although the observed interaction profiles were variable across parasite species and life stages, and no single combination demonstrated consistent synergism against all tested parasites, the overall findings support the potential of emodepside as a promising combination partner for STH treatment. Furthermore, *in vitro* cytochrome P450 inhibition assessment did not reveal clinically relevant DDIs, indicating that co-administration is unlikely to result in CYP-mediated interactions.

Future studies should focus on validating the most promising combinations *in vivo*, with priority given to those demonstrating consistent synergism across multiple species and life stages and involving drugs already deployed in clinical practice or mass drug administration programs, to maximize translational relevance.

## Data Availability

All data generated or analysed during this study are included in the article and its Supplemental material.

## SUPPLEMENTAL MATERIAL

Supplemental material aac.00825-26-s0001.docx Tables S1 to S8; Fig. S1 to S4.

ASM does not own the copyrights to Supplemental Material that may be linked to, or accessed through, an article. The authors have granted ASM a non-exclusive, world-wide license to publish the Supplemental Material files. Please contact the corresponding author directly for reuse.
