# Supplementary material for: In vitro in-depth characterization of emodepside in combination with key anthelmintic drugs on soil-transmitted helminths
Source: Antimicrob Agents Chemother. 2026 Oct 8;70(11):e00825-26. doi: 10.1128/aac.00825-26 (PMC13731180; doi:10.1128/aac.00825-26)
Supplement: Supplementary file 1 [file aac.00825-26-s0001.docx]

Supplementary Material

Table 1: Determined EC_50_ (µM ± [SE]) values of selected anthelmintics against different STH larval stages 72 hours post-incubation

| **Drugs** | ***H.polygyrus*** | ***N. americanus*** | ***A. ceylanicum*** | ***T. muris*** | ***S. ratti*** |
| --- | --- | --- | --- | --- | --- |
| Albendazole | >100 | >100 | >100 | >100 | >100 |
| Mebendazole | >100 | >100 | >100 | >100 | >100 |
| Albendazole sulfoxide | >100 | >100 | >100 | >100 | >100 |
| Abamectin | 11.0±83 | 0.3±0.02 | 0.06±0.004 | >100 | 0.05±0.006 |
| Ivermectin | 12.0±2 | 0.2±0.03 | 0.3±0.04 | >100 | 0.03±0.009 |
| Moxidectin | 0.3±0.06 | 0.2±0.02 | 0.04±0.005 | >100 | 0.01±0.002 |
| Emodepside | 1.0±0.3 | 0.4±0.03 | 2.0±0.5 | 0.3 ±0.04 | 0.5±0.02 |
| Levamisole | 2.0±0.5 | 10.4±0.1 | 20.8±0.2 | >100 | 0.5±0.002 |
| Oxantel pamoate | >100 | >100 | >100 | 4.6±1.3 | >100 |
| Pyrantel pamoate | 5.0±1.6 | 19.0±5.7 | >100 | 19.3±3.2 | 4.5±2.3 |
| Tribendimidine | >100 | 4.4±0.1 | 18.6±1.1 | >100 | 0.7±0.05 |

SE: standard error

Table 2: Determined EC₅₀ (µM ± [SE]) values for emodepside against STH adult stage at 24, 48, and 72 hours post-incubation

| **Drugs [µM]** | ***H.polygyrus*** | | | ***T. muris*** | | | ***S. ratti*** | | |
| --- | --- | --- | --- | --- | --- | --- | --- | --- | --- |
|  | 24 | 48 | 72 | 24 | 48 | 72 | 24 | 48 | 72 |
| Emodepside | 0.1±0.04 | 0.2±0.02 | 0.1±0.01 | 0.01±0.002 | 0.009±0.008 | 0.01±0.006 | 0.4±0.09 | 0.3±0.1 | 0.3±0.04 |

SE: standard error

Figure 1: Isobolograms illustrating the interaction of emodepside in combination with partner drugs at three fixed ratios (3:1, 2:2, 1:3; emodepside:partner drug (v:v)) against (A) larval and (B) adult stages of different STH models. The error bars represent the SEM (standard error of the mean).

Table 3: Individual fractional inhibitory concentration (FIC ± [SEM]) values for emodepside in combination with partner drugs at three fixed ratios (3:1, 2:2, 1:3 emodepside:partner drug (v:v)) against STH larval stages. All FIC values were calculated based on EC_50_ values determined 72 hours post-incubation.

|  | | ***H.polygyrus*** | | ***N. americanus*** | | ***A. ceylanicum*** | | ***T. muris*** | | ***S. ratti*** | |
| --- | --- | --- | --- | --- | --- | --- | --- | --- | --- | --- | --- |
| **Partner Drug** | **Ratio** | FIC_A_ | FIC_B_ | FIC_A_ | FIC_B_ | FIC_A_ | FIC_B_ | FIC_A_ | FIC_B_ | FIC_A_ | FIC_B_ |
| Abamectin | 1:3 | 0.02 [0.01] | 0.10 [0.01] | 0.41 [0.25] | 0.41[0.37] | 0.79 [0.15] | 1.13 [0.38] | * | * | 0.22 [0.12] | 0.37[0.23] |
|  | 2:2 | 0.03 [0.01] | 0.05 [0.02] | 0.64 [0.32] | 1.07 [0.79] | 1.19 [0.48] | 0.54 [0.29] | * | * | 0.57 [0.27] | 0.27[0.23] |
|  | 3:1 | 0.02 [0.01] | 0.04 [0.03] | 0.63 [0.51] | 1.07 [0.79] | 1.13 [0.70] | 0.23 [0.10] | * | * | 0.67 [0.17] | 0.13[0.07] |
| Ivermectin | 1:3 | 0.04 [0.02] | 0.52 [0.15] | 0.29 [0.01] | 1.39 [0.61] | 0.44 [0.2] | 0.43 [0.23] | * | * | 0.22 [0.08] | 1.63[0.38] |
|  | 2:2 | 0.12 [0.05] | 0.27 [0.1] | 0.26 [0.24] | 0.51 [0.49] | 0.47 [0.13] | 0.31 [0.16] | * | * | 0.33 [0.07] | 0.89[0.01] |
|  | 3:1 | 0.35 [0.18] | 0.23 [0.1] | 1.11 [0.11] | 0.61 [0.19] | 0.95 [0.28] | 0.23 [0.09] | * | * | 0.62 [0.08] | 0.56[0.06] |
| Moxidectin | 1:3 | 0.4 [0.32] | 2.13 [1.07] | 0.26 [0.07] | 1.47 [0.55] | 0.24 [0.05] | 0.41 [0.18] | * | * | 0.63 [0.30] | 3.17[1.94] |
|  | 2:2 | 0.17 [0.05] | 0.53 [0.27] | 0.35 [0.02] | 0.67 [0.13] | 0.70 [0.16] | 0.45 [0.14] | * | * | 0.87 [0.41] | 1.33[0.85] |
|  | 3:1 | 0.24 [0.04] | 0.30 [0.19] | 0.32 [0.10] | 0.56 [0.28] | 0.97 [0.21] | 0.52 [0.24] | * | * | 0.64 [0.35] | 0.37[0.27] |
| Levamisole | 1:3 | 0.34 [0.04] | 1.45 [0.55] | * | * | * | * | * | * | 0.67 [0.27] | 3.01[0.83] |
|  | 2:2 | 0.60 [0.40] | 0.85 [0.75] | * | * | * | * | * | * | 0.57 [0.12] | 0.88 [0.07] |
|  | 3:1 | 1.06 [0.56] | 0.52 [0.38] | * | * | * | * | * | * | 0.78 [0.23] | 0.38 [0.03] |
| Oxantel pamoate | 1:3 | * | * | * | * | * | * | 0.16 [0.13] | 0.49 [0.43] | * | * |
|  | 2:2 | * | * | * | * | * | * | 0.18 [0.07] | 0.15 [0.06] | * | * |
|  | 3:1 | * | * | * | * | * | * | 0.60 [0.55] | 0.16 [0.16] | * | * |
| Pyrantel pamoate | 1:3 | 0.14 [0.06] | 0.40 [0.09] | * | * | * | * | 0.45 [0.30] | 0.64[0.40] | 0.37 [0.03] | 0.83 [0.13] |
|  | 2:2 | 0.58 [0.24] | 0.53 [0.05] | * | * | * | * | 0.59 [0.16] | 0.27 [0.10] | 0.60 [0.0] | 0.44 [0.12] |
|  | 3:1 | 0.58 [0.24] | 0.32 [0.01] | * | * | * | * | 1.16 [0.59] | 0.18 [0.11] | 0.68 [0.22] | 0.16 [0.0] |
| Tribendimidine | 1:3 | * | * | * | * | * | * | * | * | 0.18 [0.12] | 0.78 [0.34] |
|  | 2:2 | * | * | * | * | * | * | * | * | 0.65 [0.45] | 0.87 [0.46] |
|  | 3:1 | * | * | * | * | * | * | * | * | 0.83 [0.57] | 0.39 [0.20] |

SEM: standard error of the mean

* see Figure 2, SF2 and ST5

Table 4: Individual fractional inhibitory concentration (FIC ± [SEM]) values for emodepside in combination with partner drugs at three fixed ratios (3:1, 2:2, 1:3 emodepside:partner drug (v:v)) against adult stage of different STH models. All FIC values were calculated based on EC_50_ values determined 72 hours post-incubation.

|  | | ***H.polygyrus*** | | ***T. muris*** | | ***S. ratti*** | |
| --- | --- | --- | --- | --- | --- | --- | --- |
| **Partner Drug** | **Ratio** | FIC_A_ | FIC_B_ | FIC_A_ | FIC_B_ | FIC_A_ | FIC_B_ |
| Albendazole | 1:3 | * | * | 0.24 [0.04] | 0.34 [0.22] | n.a | n.a. |
|  | 2:2 | * | * | 0.51 [0.09] | 0.26 [0.2] | n.a. | n.a. |
|  | 3:1 | * | * | 0.8 [0.2] | 0.14 [0.09] | n.a. | n.a. |
| Mebendazole | 1:3 | * | * | 0.29 [0.35] | 1.07 [1.60] | n.a. | n.a. |
|  | 2:2 | * | * | 0.50 [0.49] | 0.62 [0.67] | n.a. | n.a. |
|  | 3:1 | * | * | 0.48 [0.58] | 0.20 [0.31] | n.a. | n.a. |
| Albendazole sulfoxide** | 1:3 | * | * | 0.15 | 0.17 | n.a. | n.a. |
|  | 2:2 | * | * | 0.40 | 0.18 | n.a. | n.a. |
|  | 3:1 | * | * | 0.25 | 0.04 | n.a. | n.a. |
| Ivermectin | 1:3 | * | * | 0.28 [0.03] | 0.63 [0.41] | 0.50 [0.25] | 1.25 [0.75] |
|  | 2:2 | * | * | 0.18 [0.13] | 0.19 [0.21] | 0.79 [0.34] | 0.68 [0.23] |
|  | 3:1 | * | * | 0.71 [0.09] | 0.20 [0.14] | 0.88 [0.38] | 0.30 [0.1] |
| Moxidectin** | 1:3 | * | * | 0.05 | 0.52 | 0.26 [0.19] | 0.43 [0.21] |
|  | 2:2 | * | * | 0.10 | 0.27 | 0.70 [0.50] | 0.29 [0.14] |
|  | 3:1 | * | * | 0.15 | 0.18 | 0.56 [0.06] | 0.13 [0.04] |
| Levamisole** | 1:3 | 0.35 [0.19] | 0.67 [0.17] | 0.25 | 0.93 | n.a. | n.a. |
|  | 2:2 | 0.55 [0.14] | 0.33 [0.04] | 0.45 | 0.83 | n.a. | n.a. |
|  | 3:1 | 1.00 [0.11] | 0.25 [0.05] | 0.15 | 0.26 | n.a. | n.a. |
| Oxantel pamoate | 1:3 | * | * | 0.14 [0.12] | 0.96 [0.61] | n.a. | n.a. |
|  | 2:2 | * | * | 0.21 [0.13] | 0.64 [0.47] | n.a. | n.a. |
|  | 3:1 | * | * | 0.48 [0.30] | 0.47 [0.36] | n.a. | n.a. |
| Pyrantel pamoate | 1:3 | 0.26 [0.20] | 0.59 [0.30] | 0.03 [0.02] | 0.60 [0.49] | n.a. | n.a. |
|  | 2:2 | 0.84 [0.17] | 0.65 [0.04] | 0.18 [0.19] | 0.37 [0.23] | n.a. | n.a. |
|  | 3:1 | 1.56 [0.15] | 0.25 [0.10] | 0.28 [0.26] | 0.27 [0.18] | n.a. | n.a. |
| Tribendimidine | 1:3 | 0.25 0.05 | 0.94 0.44 | 0.79 [0.06] | 0.66 [0.13] | n.a. | n.a. |
|  | 2:2 | 0.50 0.2 | 0.64 0.36 | 0.38 [0.06] | 0.50 [0.0] | n.a. | n.a. |
|  | 3:1 | 2.5 0.5 | 0.73 0.02 | 0.58 [0.25] | 0.25 [0.13] | n.a. | n.a. |

SEM: standard error of the mean; n.a.: not applicable

* see Figure 3, SF3 and ST7

** the FIC values were determined from one experiment only

Figure 2: Generalized linear model (GLM) predicted survival probability curves for emodepside alone and in presence of fixed concentrations (1, 5, and 10 µM) of partner drugs for larval stages of different STH models, survival data collected at 72 hours. Error bars represent 95% confidence intervals. Confidence intervals indicating unreliable estimates due to complete or near-complete mortality, are not shown. White cells indicate conditions that were not tested with this method see Table 1 and SF1.

Table 5: Determined EC_50_± [SE] values of emodepside in presence of fixed concentrations (1, 5, and 10 µM) of partner drugs against STH larval 72 hours post-incubation.

| **Partner Drug** | **[µM]** | | ***H. polygyrus*** | ***A. ceylanicum*** | ***N. americanus*** | | ***T. muris*** | ***S. ratti*** |
| --- | --- | --- | --- | --- | --- | --- | --- | --- |
| Albendazole | | 1 | 1.5 [0.3] | 0.6[0.34] | 0.3 [0.05] | | 0.6 [0.06] | 0.5 [0.08] |
|  |  | 5 | 1.1 [0.4] | 0.3 [0.2] | 0.4 [0.09] | | 0.4 [0.08] | 0.54 [0.0] |
|  |  | 10 | 1.7 [0.3] | 0.2 [0.06] | 1.1 [0.2] | | 0.3 [0.02] | 0.2 [0.01 |
| Mebendazole | | 1 | 1.2 [0.1] | 0.6 [0.5] | 0.2 [0.03] | | 0.8 [0.02] | 0.4 [0.05] |
|  |  | 5 | 1.3 [0.3] | 0.3 [0.1] | 0.3 [0.05] | | 0.6 [0.06] | 0.2 [0.2] |
|  |  | 10 | 1.6 [0.3] | 0.3 [0.02] | 0.3 [0.05] | 0.5 [0.05] | | 0.1 [0.007] |
| Albendazole sulfoxide | | 1 | 1.9 [0.4] | 0.3 [0.3] | 1.0 [1.9] | 0.7 [0.2] | | 0.3 [0.09] |
|  |  | 5 | 1.7 [0.2] | 0.1 [0.03] | 6.5 [2.8] | 0.4 [0.01] | | 0.1 [0.01] |
|  |  | 10 | 1.5 [0.2] | 0.1 [0.01] | 4.0 [1.5] | 0.5 [0.03] | | 0.2 [0.04] |
| Ivermectin | | 1 | * | * | * | 0.3 [0.05] | | * |
|  |  | 5 |  |  |  | 0.2 [0.02] | |  |
|  |  | 10 |  |  |  | 0.2 [0.03] | |  |
| Abamectin | | 1 | * | * | * | 0.4 [0.05] | | * |
|  |  | 5 |  |  |  | 0.6 [0.19 | |  |
|  |  | 10 |  |  |  | 0.3 [0.06] | |  |
| Moxidectin | | 1 | * | * | * | 0.7 [0.06] | | * |
|  |  | 5 |  |  |  | 0.4 [0.08] | |  |
|  |  | 10 |  |  |  | 0.4 [0.09] | |  |
| Levamisole | | 1 | * | 1.2 [0.4] | 0.6 [0.3] | 1.0 [0.3] | | * |
|  |  | 5 |  | 0.7 [0.07 | 0.04 [0.06] | 0.7 [0.1] | |  |
|  |  | 10 |  | 0.1 [0.01] | 0.01 [0.01] | 0.5 [0.03] | |  |
| Oxantel pamoate | | 1 | 0.2 [0.09] | 2.2 [0.6] | 0.3 [0.06] | * | | 0.1 [0.02] |
|  |  | 5 | 0.2 [0.03] | 0.4 [0.05] | 0.2 [0.01 |  |  | 0.1 [0.01] |
|  |  | 10 | 0.1 [0.03] | 0.1 [0.02] | 0.1 [0.01] |  |  | 0.04 [0.005] |
| Pyrantel pamoate | | 1 | * | 0.6 [0.1] | 0.7 [0.05] | * | | 0.8 [0.03] |
|  |  | 5 |  | 0.7 [0.1] | 1.3 [0.3] |  |  | 0.4 [0.09] |
|  |  | 10 |  | 0.5 [0.1] | 7.8 [0.7] |  |  | 0.3 [0.04] |
| Tribendimidine | | 1 | 0.3 [0.2] | 0.6 [0.4] | 0.2 [0.06] | 0.3 [0.05] | | * |
|  |  | 5 | 0.1 [0.04] | 0.4 [0.03] | 0.2 [0.03] | 0.3 [0.1] | |  |
|  |  | 10 | 0.06 [0.003] | 0.2[0.04] | 0.1 [0.01 | 0.1 [0.006] | |  |

* see Table 1, SF1A and ST3

Table 6 Fractional inhibitory concentration (FIC) index values for emodepside in combination with partner drugs at three fixed ratios (3:1, 2:2, 1:3 emodepside:partner drug (v:v)) All FIC values were calculated based on EC_50_ values determined at 24 and 48 hours post-incubation. FIC < 0.9 synergy, FIC= 0.9–1.5 additivity, FIC > 1.5 antagonism.

|  | **24h** | | | | | | | | |
| --- | --- | --- | --- | --- | --- | --- | --- | --- | --- |
| **Partner Drugs** | ***H. polygyrus*** | | | ***T. muris*** | | | ***S. ratti*** | | |
|  | 3:1 | 2:2 | 1:3 | 3:1 | 2:2 | 1:3 | 3:1 | 2:2 | 1:3 |
| Albendazole | * | | | n.d | n.d. | n.d. | n.a. | | |
| Mebendazole | * | | | 0.67 | 0.68 | n.d. | n.a. | | |
| Albendazole sulfoxide | * | | | 0.44 | 0.56 | 0.35 | n.a. | | |
| Abamectin | n.a. | | | n.a. | | | * | | |
| Ivermectin | * | | | 0.79 | 0.49 | 0.69 | 1.42 | 1.04 | 0.77 |
| Moxidectin | * | | | 0.35 | 0.32 | 0.32 | 1.12 | 1.65 | 1.23 |
| Levamisole | n.d | n.d | n.d | 0.37 | 1.77 | 1.07 | n.a. | | |
| Oxantel pamoate | * | | | 0.79 | 0.73 | 0.67 | n.a. | | |
| Pyrantel pamoate | n.d | n.d | n.d | 0.15 | n.d. | n.d. | n.a. | | |
| Tribendimidine | n.d | n.d | n.d | 0.41 | 1.16 | 10.62 | n.a. | | |
|  | **48 h** | | | | | | | | |
| Albendazole | * | | | 1.58 | 1.96 | 1.59 | n.a. | | |
| Mebendazole | * | | | 1.13 | 0.71 | 0.43 | n.a. | | |
| Albendazole sulfoxide | * | | | 0.6 | n.d. | 0.84 | n.a. | | |
| Abamectin | n.a. | | | n.a. | | | * | | |
| Ivermectin | * | | | 0.77 | 0.71 | 1.44 | 1.78 | 1.38 | 0.98 |
| Moxidectin | * | | | n.d | n.d. | n.d. | 1.80 | 1.01 | 0.94 |
| Levamisole | 1.07 | 0.93 | 1.42 | 0.66 | 1.17 | 1.47 | n.a. | | |
| Oxantel pamoate | * | | | 0.55 | 0.33 | 0.5 | n.a. | | |
| Pyrantel pamoate | 1.10 | 1.23 | 1.88 | 0.60 | 0.35 | 0.51 | n.a. | | |
| Tribendimidine | 1.38 | 0.90 | 0.91 | 0.56 | 0.53 | 0.70 | n.a. | | |

n.a.: not applicable, n.d.: could not be determined

* see ST7

Figure 3: Dose-response curves of emodepside alone and in presence of a partner drug at fixed concentrations (1, 5, and 10 µM). The individual points are represented as mean± [SE] of individual experiments and dashed lines represent 95% confidence intervals of the fitted curve. A) adult H. polygyrus B) adult S.ratti

Table 7: Dertermined EC50± [SE] values of emodepside in presence of fixed concentrations (1, 5, and 10 µM) of partner drugs against adult stages of different STH models at 24, 48 and 72 hours post-incubation.

| **Partner Drug** | **[µM]** | ***H. polygyrus*** | | | ***S. ratti*** | | |
| --- | --- | --- | --- | --- | --- | --- | --- |
|  |  | 24 | 48 | 72 | 24 | 48 | 72 |
| Albendazole | 1 | 0.05 [0.01] | 0.05 [0.05] | 0.04 [0.01] | n.a. | n.a. | n.a. |
|  | 5 | 0.03 [0.007] | 0.03 [0.02] | 0.03 [0.006] | n.a. | n.a. | n.a. |
|  | 10 | 0.04 [0.007] | 0.03 [0.06] | 0.03 [0.008] | n.a. | n.a. | n.a. |
| Mebendazole | 1 | 0.05 [0.009] | 0.04 [0.004] | 0.04 [0.5] | n.a. | n.a. | n.a. |
|  | 5 | 0.07 [0.2] | 0.03 [0.001] | 0.04 [0.9] | n.a. | n.a. | n.a. |
|  | 10 | 0.04 [0.005] | 0.04 [0.007] | 0.03 [0.005] | n.a. | n.a. | n.a. |
| Albendazole sulfoxide | 1 | 0.03 [0.006] | 0.05 [0.05] | 0.04 [0.01] | n.a. | n.a. | n.a. |
|  | 5 | 0.04 [0.003] | 0.04 [0.03] | 0.03 [0.003] | n.a. | n.a. | n.a. |
|  | 10 | 0.03 [0.05] | 0.04 [0.0008] | 0.03 [0.007] | n.a. | n.a. | n.a. |
| Ivermecine | 1 | 0.02 [4.7] | 0.03 [0.01] | 0.04 [0.005] | * | * | * |
|  | 5 | 0.03 [0.06] | 0.03 [0.09] | 0.03 [0.005] | * | * | * |
|  | 10 | 0.03 [0.005] | 0.02 [0.003] | 0.05 [0.01] | * | * | * |
| Abamectin | 1 | n.a | n.a | n.a | 0.49 [0.25] | 0.39 [0.22] | 0.26 [0.03] |
|  | 5 | n.a | n.a | n.a | 0.46 [0.04] | 0.33 [1.1] | 0.25 [0.05] |
|  | 10 | n.a | n.a | n.a | 0.39 [0.07] | 0.15 [0.05] | 0.25 [0.13] |
| Moxidectin | 1 | 0.03 [0.8] | 0.02 [0.1] | 0.03 [0.04] | * | * | * |
|  | 5 | 0.02 [0.02] | 0.02 [0.01] | 0.03 [0.07] | * | * | * |
|  | 10 | 0.02 [0.006] | 0.02 [0.003] | 0.03 [0.3] | * | * | * |
| Levamisole | 1 | * | * | * | n.a. | n.a. | n.a. |
|  | 5 | * | * | * | n.a | n.a. | n.a. |
|  | 10 | * | * | * | n.a. | n.a. | n.a. |
| Oxantel pamoate | 1 | n.d.^a^ | 0.04 [0.008] | 0.02 [0.008] | n.a. | n.a. | n.a. |
|  | 5 | 0.07 [0.03] | 0.03 [0.01] | 0.04 [0.8] | n.a. | n.a. | n.a. |
|  | 10 | 0.2 [0.9] | 0.04 [0.9] | 0.02 [0.008] | n.a. | n.a. | n.a. |
| Tribendimidine | 1 | * | * | * | n.a. | n.a. | n.a. |
|  | 5 | * | * | * | n.a. | n.a. | n.a. |
|  | 10 | * | * | * | n.a. | n.a. | n.a. |

SE: standard error; n.a.: not applicable; n.d.^a^: not determined, non-monotonic dose-response observed

* see Table 2. SF1B and SF4

Table 8: Inhibitory activity of individual compounds against selected CYP450 enzymes at 100 µM, expressed as percentage inhibition (% ± SE).

| **Drugs** | **CYP 1A2** | **CYP 2C9** | **CYP 2C19** | **CYP 2D6** | **CYP 3A4** |
| --- | --- | --- | --- | --- | --- |
| Emodepside | 0±0 | 57.3±2.9 | 0±0 | 37.9±4.7 | 99.1±1.6 |
| Levamisole | 23.7±4.6 | 16.1±2.0 | 10.6±1.5 | 81.7±0.2 | 45.2±15.6 |
| Oxantel pamoate | 46.1±0.6 | 83.3±3.8 | 69.6±4.1 | 93.7±0.4 | 56.2±34.4 |
| Pyrantel pamoate | 64.4±4.7 | 75.0±6.3 | 46.0±1.6 | 93.0±0.3 | 40.2±17.2 |
| Tribendimidine | 3.43* | 14.4±9.9 | 7.5±0.8 | 52.6±0.2 | 33.7±19.4 |
| Albendazole | 57.1±1.8 | 21.8±6.7 | 0±0 | 14.0±3.7 | 10.2±2.8 |
| Mebendazole | 70.7±14.4 | 81.0±2.0 | 20.7±7.7 | 31.1±2.3 | 0±0 |
| Albendazole sulfoxide | 84.7±2.3 | 38.4±1.6 | 15.6±3.4 | 0±0 | 40.6±16.4 |
| Ivermectin | 0±0 | 99.9±2.8 | 0.6±0.8 | 89.8±1.0 | 100 |
| Moxidectin | 0±0 | 100.6±0.7 | 49.6±20.2 | 72.3±10.6 | 29.1±16.4 |

SE: standard error

* only one measurement was done, due to not enough testing material being available

Figure 4: Statistical evaluation of the inhibition percentage of selected combinations against A) CYP1A2, B) CYP2C9, C) CYP2C19, D) CYP2D6, E) CYP3A4. Each panel shows a comparison of the tested combination (yellow) with emodepside (black) and parter drug (red, if applicable) across the tested concentrations. Data are represented as the mean inhibition% ± SEM (standard error of the mean) with asterisks indicating significant differences (*p<0.05, **p<0.01, ***p<0.001).
